# Species- and sex-specific chemical composition from an internal gland-like tissue of an African frog family

**DOI:** 10.1098/rspb.2023.1693

**Published:** 2024-01-10

**Authors:** Marvin Schäfer, David Sydow, Maria Schauer, Joseph Doumbia, Thomas Schmitt, Mark-Oliver Rödel

**Affiliations:** ^1^ Museum für Naturkunde – Leibniz Institute for Evolution and Biodiversity Science, Invalidenstraße 43, 10115 Berlin, Germany; ^2^ Zoology III Department of Animal Ecology and Tropical Biology, University of Würzburg, Am Hubland, 97074 Würzburg, Germany; ^3^ ONG EnviSud Guinée, Quartier Kipé T2 commune de Ratoma, 530 BP 558 Conakry, Guinea

**Keywords:** anuran, breeding gland, chemical communication, fat tissue, multimodal communication, Odontobatrachidae

## Abstract

Intraspecific chemical communication in frogs is understudied and the few published cases are limited to externally visible and male-specific breeding glands. Frogs of the family Odontobatrachidae, a West African endemic complex of five morphologically cryptic species, have large, fatty gland-like strands along their lower mandible. We investigated the general anatomy of this gland-like strand and analysed its chemical composition. We found the strand to be present in males and females of all species. The strand varies in markedness, with well-developed strands usually found in reproductively active individuals. The strands are situated under particularly thin skin sections, the vocal sac in male frogs and a respective area in females. Gas-chromatography/mass spectrometry and multivariate analysis revealed that the strands contain sex- and species-specific chemical profiles, which are consistent across geographically distant populations. The profiles varied between reproductive and non-reproductive individuals. These results indicate that the mandibular strands in the Odontobatrachidae comprise a so far overlooked structure (potentially a gland) that most likely plays a role in the mating and/or breeding behaviour of the five *Odontobatrachus* species. Our results highlight the relevance of multimodal signalling in anurans, and indicate that chemical communication in frogs may not be restricted to sexually dimorphic, apparent skin glands.

## 1. Introduction

While distinct courtship behaviour and the possession of conspicuous skin glands have drawn early attention to chemical communication in salamanders and newts, the possibility of chemical communication has been widely neglected in frogs and toads [1]. By contrast, the remarkable acoustic communication in anurans led to the belief that it is the only relevant form of communication in these animals. In more recent years, however, several publications reported chemical cues from anurans and, in some cases, even provided evidence for their use as pheromones [2–5]. Chemical communication in frogs is still widely unexplored. Most detected or suspected cases of anuran chemical communication are based on externally visible macro-glands, labelled ‘breeding glands’, which protrude during the breeding season and are restricted to male anurans [1,6]. Published examples include nuptial pads (e.g. *Rana temporaria*; [7]), femoral glands (Mantellidae; [8]) and glandular patches on the vocal sac (Hyperoliidae; [5]). Chemical signalling in anurans of both sexes includes intraspecific, e.g. kin recognition within Leiopelmatidae [9], and interspecific communication, e.g. mosquito repelling in the genus *Litoria* [10], or appeasement allomones towards ants by *Phrynomantis microps* [11]. The later examples are not limited to obvious, male-specific macro-glands, but originate from the skin. Thus, generally, chemical cues originate from exocrine, cutaneous skin glands that either produce and/or emit semiochemicals directly, or support symbiotic microorganisms in doing so [12].

A large variety of substance classes have been identified from frog skin glands thus far [13]. However, only a few classes have been associated with a signalling function. This includes volatile substances such as macrolides and terpenoids [5,14], as well as non-volatiles like oligopeptides and peptides [7,15]. Fatty acid esters are known to be found within macro-glands and on frog skin, but have not yet been assigned as signalling molecules in vertebrates [16].

During systematic research on the frog family Odontobatrachidae, endemic to the rainforests of West Africa [17], we discovered a yellow, glandular strand along the mandibular bones. A first gas chromatography/mass spectrometry (GC/MS) analysis suggested fatty acid esters as components unique to this strand. Herein, we investigate the histology and anatomy of this strand, analyze and compare the chemical profiles of the strand's secretion in all five species of the family [18] and discuss its potential signalling function and identity as a gland.

## 2. Material and methods

### (a) Collection of specimens

From December 2017 to July 2021, we collected specimens of all five *Odontobatrachus* species, *O. arndti*, *O. fouta*, *O. natator*, *O. smithi* and *O. ziama*. We euthanized, killed and dissected the animals to obtain tissue samples for chemical analysis. Tissue samples where then extracted in dichloromethane and analysed by GC/MS. To search for sex- and species-specific differences in strand composition, we sampled both sexes of each species from at least two locations per species [18]. We also sampled *O. arndti* under wet and dry season conditions on the same river, to screen for seasonal effects. To check for the effect of geographical distance between populations, we sampled *O. arndti* males and *O. smithi* females over a large part of their distribution range. To investigate ontogenetic effects on strand composition, we sampled metamorphic and subadult *O. ziama* and compared them to sexually mature animals. For histological and anatomical analyses, we used voucher specimens from the collection of the Museum für Naturkunde Berlin (ZMB). Studied animals are listed in table 1 and the electronic supplementary material (table S1); sample locations are illustrated in the electronic supplementary material (figure S1).

**Table 1.** Species, sample size, location and study season of all analysed *Odontobatrachus* specimens for the respective analysis (chemical composition with respect to species, sex and life stage, season, or geographic distance between populations; compare text). Table details: given is the total number of animals in bold, followed by sample sizes for female, juvenile and male frogs [total (female|juvenile|male)].

species	river	GPS coordinates		analysis					
		latitude	longitude	species & sex & life stage		season		geographical distance	
				dry season	dry season	dry season	wet season	dry season	wet season
*O. arndti* (*n* = 30)	Zougué I	7.69642	−8.40005	**7** (3|—|4)		**7** (3|—|4)	**9** (3|—|6)		**6** (—|—|6)
	Zougué II	7.67613	−8.38401	**4** (2|—|2)		**4** (2|—|2)	**4** (1|—|3)		
	Madey	7.64890	−8.42347						**4** (—|—|4)
	Wolanda	7.64645	−8.36702						**2** (—|—|2)
*O. fouta* (*n* = 10)	Chute de Saala	11.29389	−12.50178	**7** (3|—|4)					
	Ditiwol	10.82138	−12.19296	**3** (1|—|2)					
*O. natator* (*n* = 9)	Soyah	10.29917	−11.94376	**4** (2|—|2)					
	Wosai	8.35160	−9.41609	**5** (2|—|3)					
*O. smithi* (*n* = 11)	Ideta	10.92233	−13.15638	**3** (2|—|1)				**2** (2|—|—)	
	Naremba	10.83323	−13.83130	**6** (4|—|2)				**4** (4|—|—)	
	Sigita	10.85960	−12.55760					**2** (2|—|—)	
*O. ziama* (*n* = 40)	Vérè I	8.35732	−9.30069	**7** (4|—|3)	**36** (15|7|14)				
	Vérè II	8.34770	−9.30089	**4** (2|—|2)					
total	(*n* = 100)			**50** (26|—|24)	**36** (15|7|14)	**11** (5|—|6)	**13** (4|—|9)	**8** (8|—|—)	**10** (—|—|10)

### (b) Frog handling and sample processing

All animal handling and procedures on animals were conducted in accordance with the national standards of Germany (Tierschutzgesetz) and conducted under the authority of the Ministère de l'Enseignement Supérieur et de la Recherche Scientifique (MESRS) of the Republic of Guinea. Research, collection and export permits N°054/MESRS/DGERSIT/2018, N° 006/MESRS/DGERIST/2019 and N° 081/MESRS/DGERIST/2021 had been granted accordingly. Frogs were collected during the night and measured on site before being kept singly in plastic bags. Weight was measured with a spring balance (range 0–100 g, precision ± 0.5 g) and body length was taken as snout–vent length (SVL), using a digital caliper (± 0.1 mm). Frogs were brought to the field laboratory and stored on ice for at least 45 min [19]. Anaesthetized frogs were then killed by an injection of 0.5 ml 96% ethanol (equivalent of 10–20 g kg^−1^ bodyweight) into the brain (punctured through the foramen magnum). This procedure was necessary, as bathing or other external or internal application of anesthetic drugs (e.g. MS-222, Benzocain) would likely have resulted in contamination of strand tissues [20]. Before dissection, death of specimens was confirmed by testing for the absence of corneal reflexes as well as respiratory and cardiac arrest [21]. For dissection, specimens were placed on their dorsal side with the snout towards the preparator. The gular skin was opened and folded back, using a separate set of instruments than for the actual strand dissection to prevent contamination. To ensure standardized sampling, especially in animals with less developed strands, we dissected only the anterior, recognizable part of the strand, running parallel to the mandibular bone (apical tip roughly to the interhyoid muscle; see below). For strand extraction, the anterior tip of the fatty strand was lifted with forceps and carefully freed from the surrounding connective and muscle tissue with micro-scissors. Whenever possible we dissected the strands on both sides of the jaw and extracted them individually. This was initially done to create redundancy for the subsequent analysis, however, this also allowed us to compare strands from opposing sites. In well-developed strands, we occasionally sampled the posterior part to compare the chemical composition of the two portions. Preliminary analysis had already revealed that the strand had a unique chemical composition not found in any other body tissue (Schmitt & Rödel 2016, unpubl. data). We further verified this finding by testing tissues known to contain fat cells from a male and female *O. natator* specimen, respectively. Specifically, we sampled skin from the back, thighs and lower jaw, with varying amounts of subdermal fat tissues (trunk versus extremities; [22]). Additionally, we sampled fat tissue aggregation in the back of both sexes, along with intestinal fat bodies and liver tissue. Lastly, to assess detectability of substances from glandular fatty strands in adjacent tissue, we also sampled muscle tissue and external vocal sac skin from males and corresponding skin sections in females. Control tissues were always sampled before preparing the actual strand in order to exclude the risk of contamination. All tissue samples were placed in 1 ml PTFA sealed GC vials, filled with 1 ml dichloromethane. Tissues were removed after two hours with cleaned forceps and stored in 75% ethanol. Vials were brought to the University of Würzburg and stored at −20°C until analysis.

### (c) Gas chromatography–mass spectrometry analysis

Extracted tissue samples were analysed with a gas chromatograph coupled to a mass spectrometer (GC/MS) (GC: 5975C, MS: 6890 inert XL, Agilent Technologies, Waldbronn, Germany). A DB-5 silica column (30 m × 0.25 mm, diameter = 0.25 µm; J&W Scientific, Folsom, USA) was installed and helium was used as carrier gas at a continuous flow rate of 1 mL per minute. One µL of each sample was injected in splitless mode with an injector temperature of 300°C. The GC temperature programme started with 60°C for 1 min, increased by 5°C per minute until reaching 300°C and was maintained for 10 min. The transfer line temperature was 300°C. Electron ionization mass spectra were recorded from *m/z* 40 to 650. We used the MSD ChemStation F.01.00.1903 for data recording and analysis and the commercially available spectra library NIST 17 to tentatively identify compounds on a substance class level (library-suggested compound identities, where possible to elucidate, are given in the electronic supplementary material (table S2)). Peaks of the chromatograms were integrated and areas were extracted into a spreadsheet. Peaks of all samples were aligned following Ottensmann *et al*. [23] according to retention indices and mass spectra similarity and were log + 1 transformed to account for potential mean variance effects [24]. Initial comparisons of the chemical profiles of all tissue samples revealed a characteristic cholesterol peak and several undetermined derivatives around retention times of about 43–46 min, present in all samples across all species and usually the most abundant substances. We concluded that this fraction contained tissue-unspecific, cellular components with high molecular masses, and thus omitted all compounds exceeding a retention time of 41 min from the subsequent analysis. The cropped dataset comprised 750 peaks. Samples were ordinated in a three-dimensional dissimilarity matrix (NMDS), using the Bray-Curtis dissimilarity index as distance measure.

### (d) Statistical analysis

To validate ordinations, we tested for group homogeneity with permutational analyses of multivariate dispersions (PERMDISP) and permutational multivariate analysis of variance (PERMANOVA) for differences in group means (10 000 permutations). For spatial analysis, we additionally performed Mantel tests to control whether there was correlation between the dissimilarity matrix and a distance matrix computed from the localities of the respective samples. To identify important decisive peaks in the chemical profiles, we trained random forests on a labelled dataset and used these models (one for sex and one for species) to predict the assignment of sex and species of an unlabelled dataset. Ordination and multivariate analysis were based on a single strand excerpt per individual; we included the redundant samples to increase the random forest dataset (resampling method). In total the dataset comprised 135 individual strand samples from adult frogs collected during dry season condition. Two thirds of these data—82 samples (37 female and 45 male)—were randomly assigned to a training dataset. The remaining 53 samples (22 female and 31 male) comprised the testing dataset. All data handling and analysis was computed with R software 4.2.1 [25], using the following packages: caret [26], dplyr [27], geodist [28], Ggplot2 [29], randomForest [30], recipes [31] and vegan [32]. Complementary statistical results are given in the electronic supplementary material (table S3). The fully reproducible code and original data are available in the electronic supplement, at https://doi.org/10.5061/dryad.t1g1jwt8f [33].

### (e) Histology and three-dimensional reconstruction

Strand anatomy appeared uniform across the genus. Nevertheless, male frogs showed a potential functional connection between the vocal sac and the strand. To assess sex-specific differences in strand anatomy, we prepared histological slides from male and female *O. arndti*, *O. fouta* and *O. natator* vouchers (electronic supplementary material, table S1). Vouchers from the ZMB collection were decapitated and the head demineralized in a buffered (pH 7.4) 25% aqueous solution of EDTA, for four weeks on a linear shaker. The softened heads were dewatered in a graded series of ethanol (70%–90%). Further dehydration and paraffin infiltration were performed with a tissue processor Leica TP1020 (Wetzlar, Germany) using Xylene as intermedium. The tissue was cut at 6 µm with a sliding microtome Leica SM2010R (Wetzlar, Germany) and mounted on slides, coated with protein glycerin and stained with H&E. For better visualization, we reconstructed a simplistic, schematic anatomical 3D model of the strand and associated structures, using Blender 3.4 software [34]. Hereby, we used digitalized slides, detailed photographs taken during the strand extractions as well as additional collection material as templates (electronic supplementary material, table S1). Anatomical nomenclature follows Elias-Costa *et al*. [35] and Engelkes *et al*. [36]. High resolution scans of male and a female slides are available in the electronic supplement.

## 3. Results

### (a) Anatomical analysis

**Figure 1.**
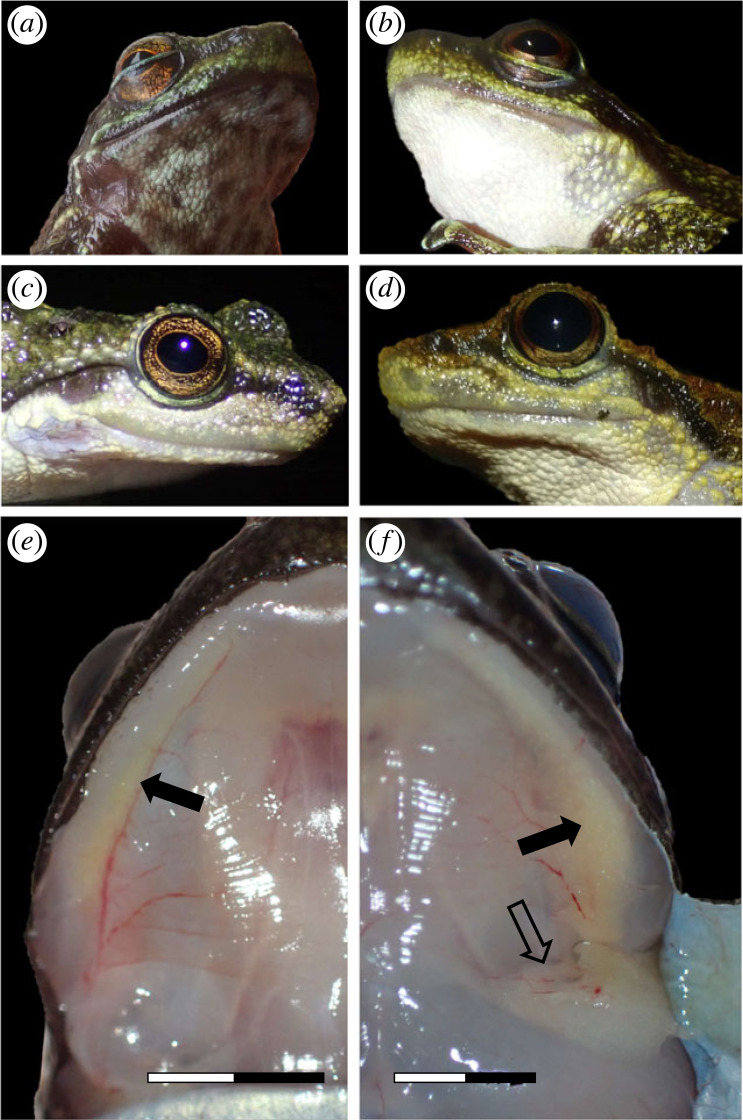
External and internal characters of the gular region in male (left column) and female (right column) *Odontobatrachus*: (*a*) reproductively active, calling *O. natator* male; (*b*) gravid *O. arndti* female; (*c*) non-calling *O. natator* male; (*d*) non-gravid *O. arndti* female. (*e*) An inconspicuous strand in a non-calling *O. arndti* male; (*f*) a well-developed strand in a gravid *O. natator* female. Scale bar, 5 mm. Arrows indicate mandibular (filled) and episternal strands (unfilled). Note: these differences applied to all *Odontobatrachus* species. Photographs by M. Schäfer.

We examined 100 frogs for chemical analysis (table 1). Dissections also allowed a macroscopic assessment of the strand–jaw–vocal sac arrangement. The strand is not externally visible, but the paired outer vocal sacs of males were conspicuous, especially in actively calling individuals. External vocal sacs appeared as dark collapsed skin flaps (figure 1*a*), and in non-calling males, they were inconspicuous with lighter colour and smooth skin (figure 1*c*). Females had a distinct externally visible skin section in a similar position as the vocal sac in males (figure 1*b*). However, this was not always obvious, especially in smaller and non-gravid females. This skin section had fewer tubercles compared to the surrounding skin and sometimes darker pigmentation, likely due to the tissue below (figure 1*d*). We hereafter refer to it as ‘post-mandibular patch’.

#### (i) Macroscopic description

Upon removal of the gular skin, two yellowish or white granular strands were visible on each side of the mandible (figure 1*e,f*). The macroscopic appearance of the strands strongly varied among individuals. In both sexes and across all species, we found individuals with inconspicuous, barely visible thin threads up to massive eye-catching granulated strands (compare figure 1*e,f*). Actively calling males and gravid females had better-developed strands, coinciding with flabby outer vocal sacs in males and widened tubercles in the females' post-mandibular patch.

**Figure 2.**
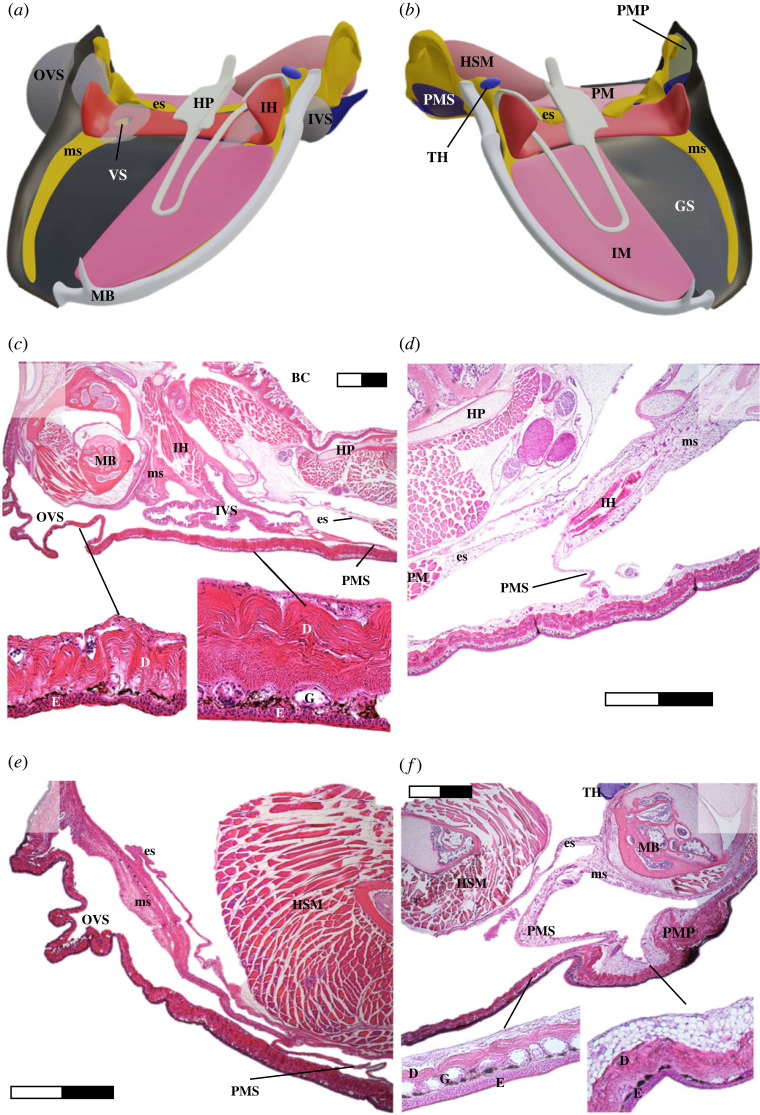
Anatomical differences of the gular region and histology of the mandibular strand in male (left column) and female (right column) *Odontobatrachus*. (*a*) Arrangement of glandular strand, vocal sac and associated musculature in a male; (*b*) same view in a female. (*c*) Strand–vocal sac interaction in a calling / reproductive *O. ziama* male (well-developed strand); (*d*) strand–skin interaction in a non-gravid *O. ziama* female (underdeveloped strand). (*e*) Histology of an underdeveloped strand in a *O. arndti* male (ZMB 78360). (*f*) Histology of a well-developed strand in a gravid *O. arndti* female (ZMB 78361). BC, buccal cavity; D, dermis; E, epidermis; es, episternal strand (yellow); G, granular skin glands; GS, gular skin (dark grey); HSM, humero-scapula musculature (pink); HP = hyoid plate (white-grey; only one half displayed; IH, interhyoidal musculature (dark red); IM, intermandibular musculature (pink; only one side displayed); IVS, inner vocal sac (transparent white; non-inflated); MB, mandibular bone (white; only one side displayed); ms, mandibular strand (yellow); OVS, outer vocal sac (transparent white; inflated); PM, pectoral musculature (pink); PMS, post-mandibular septum (dark blue); PMP, post-mandibular patch (light grey); TH, thymus (light blue); VS, vocal sac. Scale bar, 1 mm. Note: compare size of macrocytic fat cells in subdermal fat tissue and mandibular strand in (*f*). Photographs and illustrations by M. Schäfer.

A large anterior (cranial) strand originates at the level of the mandibular symphysis and runs parallel to the mandibular bone, extending just posteriorly (dorsal) to the mandibular joint. We hereafter refer to it as ‘mandibular strand’. During dissection, it had to be separated from the mandible bone and the intermandibular musculature, but its dorsal margin appeared unconnected to underlying tissue. The mandibular strand appeared smooth and sharply demarcated from surrounding tissue for most of its course, but became slightly irregular in shape further posteriorly and was directly attached to the interhyoid muscle (figure 1*f*). Posteriorly, at the pectoral sinus, it dived down dorsally, reaching the thymus. At its posterior end, the mandibular strand fused with the skin, forming a small pocket. This contact point corresponded to the males' outer vocal sac and the females’ post-mandibular patch. Medial to this connection, the mandibular strand merged with a second, shorter strand originating at the level of the omosternum. This strand was well attached posterior-medially to the episterno-humeral muscle, with its medial tip reaching underneath the interhyoid muscle. We therefore refer to it as ‘episternal strand’ (figure 2). The dorsal margin of the episternal strand laid loosely on top of the humero-scapula muscle, while its anterior-cranial margin (apart from the medial tip) merged with the post-mandibular septum. This septum, separating the submandibular and pectoral lymphatic sacs, originated at the posterior margin of the interhyoid muscle and fused postero-laterally with the skin (including the post-mandibular patch and the outer vocal sac). The septum thus covered the episternal strand, resulting in a duller appearance when compared to the mandibular strand (figures 1*f* and 2*c*).

While this general anatomy applied to both sexes, we noted sex-specific anatomical differences along the episternal strand, post-mandibular septum and skin. In females, the septum appeared hypertrophied and particularly the lateral portion (fusing with the post-mandibular patch) was well interlaced with episternal fatty strand tissue. In males, the septum was thinner, lacked fatty strand tissue and passed over the inner vocal sac before reaching the skin (including outer vocal sac). This difference became particularly evident upon mechanical manipulation. When lifting the females' post-mandibular patch, the septum automatically stretched open (figure 2*d*) while in males it immediately tore apart when lifting the outer vocal sac (figure 2*c*). Presumably, these differences are due to the vocal sac anatomy, as a strengthened septum would hinder it from inflating [37].

#### (ii) Histological description

We prepared eight individuals for histological analysis: one male and one female each of *O. arndti* and *O. ziama*, along with four juvenile *O. fouta* (electronic supplementary material, table S1). In cross-section, well-developed mandibular strands appeared homogeneously spongy, mainly consisting of swollen macro-vesicular fat tissue. Underdeveloped strands only comprised remaining, collapsed fibroblasts, but revealed that the strands’ extracellular matrix consists only of elastic fibres, lacking interlaced muscle fibres, and a surrounding layer of collagenous connective tissue (figure 2*e,f*). Irrespective of the developmental stage, a large artery and several veins as well as a side-branch of the trigeminus nerve, entering from the pectoral sinus, supplied the strand along its entire anterior length (figure 2*e*,*f*). At the level of the intermandibular muscle, the strand was never directly attached to the muscle, but fixated between muscle and mandibular bone via connective tissue. Thereby the cranial margin, which was not connected to any tissue, formed a small sinus between strand, bone and buccal floor (figure 2*e*,*f*). By contrast, at the level of the interhyoid muscle the strand sat directly on the epimysium of the interhyoid muscle, with a more extensive and direct connection to the mandibular bone (compare mandibular strand, ms, in figure 2*e* and electronic supplementary material, figure S2A). Posteriorly, strand tissue was mainly connected to the loose connective tissue of the pectoral sinus. The pocket-like end of the mandibular strand was the only direct connection between strand tissue and the dermis of the skin in males (electronic supplementary material, figure S2C).

The internal structure of the episternal strand matched the structure of the mandibular strand, but it was less vascularized. Only a single vein was apparent, but no arteries or nerves. In both sexes, the strand was directly attached to the epimysium of the episterno-humeral muscle. However, the connection of its anterior margin differed between male and female frogs. In males, the episternal strand was not connected to the interhyoid muscle, but instead to the cranial portion of the inner vocal sac. This connection was indirect (via connective tissue) at the anterior-medial part (the medial tip of the strand reaching underneath the interhyoid muscle) but direct further posterior-laterally (electronic supplementary material, figure S2A). In females, the episternal strand was closely associated with the interhyoid muscle instead. Again, this connection was indirect via connective tissue at the anterior-medial part, while further posterior-laterally strand tissue sat directly on the epimysium of the interhyoid muscle (electronic supplementary material, figure S2). Consequently, in females, at the point where the medial tip of the episternal strand emerged from under the interhyoid muscle, the muscle is surrounded by tissue from both strands, dorsally from the episternal and ventrally from the mandibular strand. Here, the post-mandibular septum of the female increasingly interlaced fatty strand tissue, ultimately forming the robust connection to the skin further posterior-laterally (figure 1*d*; electronic supplementary material, figure S2A). This connection hence constitutes a second point of contact of strand and the skin, unique to the females (electronic supplementary material, figure S2). In males, the septum also fuses with the skin, however this never involves fat tissue; instead, the inner vocal sac is in close contact with the episternal and mandibular strand (figure 2*d*; electronic supplementary material, figure S2). Both the outer vocal sac and the post-mandibular patch showed histological differences compared to normal adjacent skin. In females, the dermis was hypertrophied and the epidermis appeared thinned out. In males, the dermis and epidermis appeared thinned out compared to normal skin, with rare or completely absent epidermal skin glands in these areas (inset figures in electronic supplementary material, figure S2A & D).

### (b) Chemical analysis

The chemical profiles of all species differed, while profiles of female and male frogs of a species were more similar to each other, compared to other species. Both sexes of a species often shared the same compounds (peaks with identical mass spectra and retention index). However, sex-specific differences in the profiles were visible (electronic supplementary material, figure S4). Particularly, shared compounds among sexes would show concentration differences, yet peaks could be fully absent in either sex. Peaks in all species clustered around a retention time of 28–35 min (electronic supplementary material, figures S4, S5 & S6). The fragmentation pattern of the mass spectra of these peaks predominantly hinted towards various esters of several fatty acids as palmitic, stearic and oleic acid, however with undetermined structures due to the lack of library matches and commercially available standards. Underdeveloped strands yielded lower abundances but had identical profiles (electronic supplementary material, figure S9). Mandibular and episternal strands also showed identical chemical profiles (electronic supplementary material, figure S3). In the control tissues from *O. natator*, the profile of the strand tissue was only discernible in the outer vocal sac in males and the post-mandibular patch in females. Other tissue and skin sections had different profiles from the controls (electronic supplementary material, figures S5 & S6).

#### (i) Species and sex comparison

**Figure 3.**
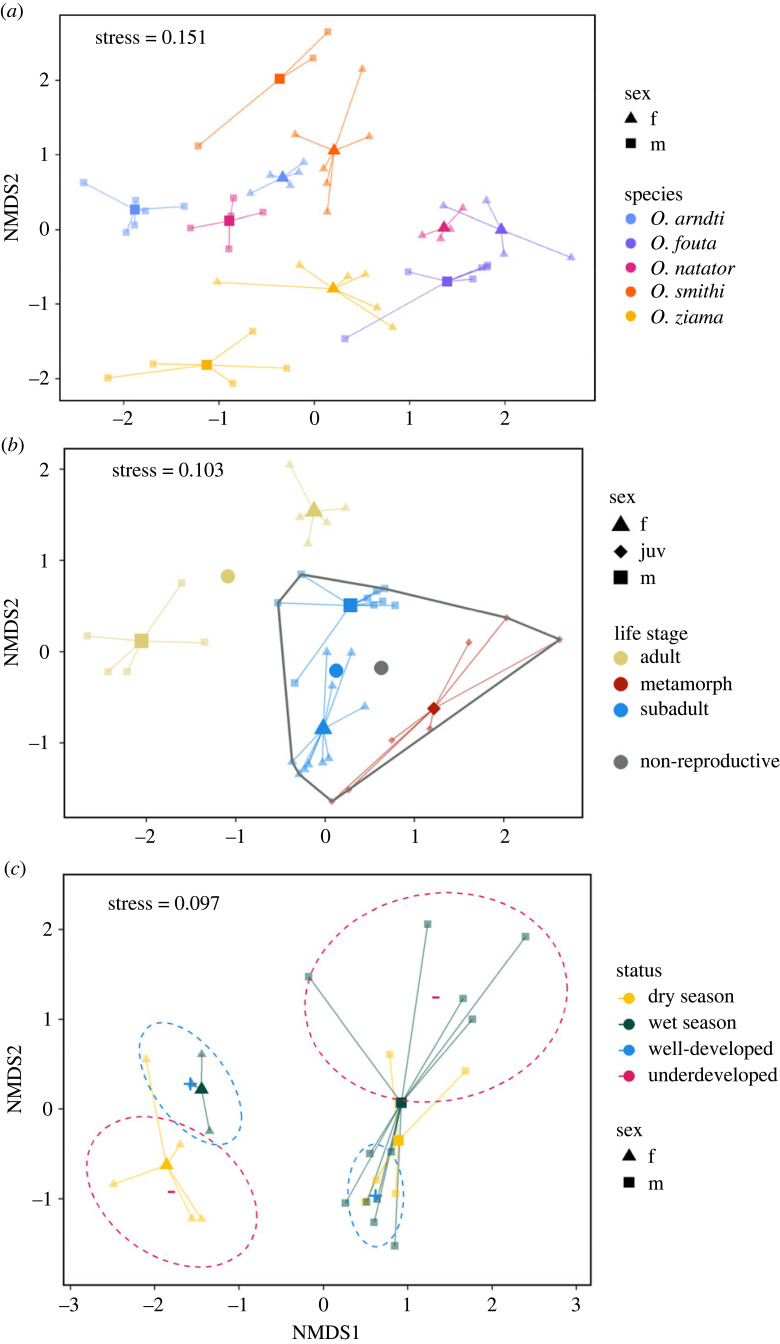
Three-dimensional non-metric multidimensional scaling (NMDS) plot of GC/MS data for three different cohorts of *Odontobatrachus* mandibular strand samples, based on Bray–Curtis similarity values. (*a*) 50 extracts from all five *Odontobatrachus* species and both sexes (*O. arndti*, *O. fouta*, *O. natator*, *O. smithi* and *O. ziama*) (compare electronic supplementary material, figure S4). (*b*) 36 extracts of *Odontobatrachus ziama* mandibular strands according to frogs' ontogeny. The grey dot refers to the group mean and the surrounding polygon highlights the samples that contribute to this mean, and hence represent all non-reproductive life stages (merged subadults und metamorphs). (*c*) 26 extracts of *O. arndti* mandibular strands according to season and strand development. Ellipses and operators indicate grouping according to developmental status of the strand (light blue colour and ‘+’ for well-developed, light red and ‘−’ for underdeveloped strands). Large symbols constitute group means calculated from the distances within group samples (small symbols). Distances and hence real dissimilarities among species and sexes are arbitrary as plot omits third dimension.

To test whether chemical composition of the strands would vary across species and sexes, we analysed a total of 50 mandibular strand samples from adult *Odontobatrachus* of both sexes (25 females, 25 males; table 1) and all five species in dry season condition. Group variability was homogeneous for species (d.f. = 4, *F* = 0.30, *p* = 0.88) and sex (d.f. = 1, *F* = 0.22, *p* = 0.64). The permutational multivariate analysis of variance indicated a clear separation of species (d.f. = 4, *F* = 2.66, *p* < 0.01) and sex (d.f. = 1, *F* = 2.34, *p* < 0.01), which was also apparent from the ordination plot (figure 3*a*).

#### (ii) Random forest analysis

To validate species- and sex-specificity, we performed a random forest analysis for both variables. The optimized random forest for species (500 trees, 14 nodes, 16 split variables = m try) achieved 92.8% accuracy (52 correct predictions out of 53 samples; Kappa = 0.95, *p* < 0.01). For sex, the optimized forest (250 trees, 10 nodes, 74 split variables) achieved 92.5% accuracy (49 correct predictions; Kappa = 0.84, *p* < 0.01). We extracted the most decisive peaks (mean accuracy) from the models, with around 550 out of 750 peaks contributing information, while 200 were irrelevant. Thirty peaks alone explained approximately one quarter of the correct predictions. These decisive peaks clustered around a retention time of 28–39 min in the chromatograms. Individual peaks were not consistently found in all samples of a specific species or sex, as they appeared in several species or sexes (table 2; electronic supplementary material, table S2). This suggests that the species and sex-specificity relied on a complex mix of multiple substances rather than single specific compounds.

**Table 2.** Retention time and substance class of the most important compounds (based on mean accuracy) identified by GC-MS in female (f) and male (m) *Odontobatrachus* mandibular strands. Table details: given are mean accuracy of compounds for assigning sex and species from the random forest analysis. Signs indicate frequency of the respective peak in all samples of both sexes for all *Odontobatrachus* species (empty cell, not detected; ∼ < 25%; + >25%, ++ > 50%, +++ > 75%).

retention time (min)			retention index	substance class	mean accuracy		*O. arndti*		*O. fouta*		*O. natator*		*O. smithi*		*O. ziama*	
mean	±	s.d.			sex	species	f	m	f	m	f	m	f	m	f	m
8.25	±	0.02	1182	aldehyde		6.64						++	++	++		∼
11.22	±	0.06	1301	aldehyde		8.35							+++	++		
15.01	±	0.09	1454	hydrocarbon		6.13		++				∼	∼			
25.88	±	0.01	1956	fatty acid ester	4.02	5.19							+++			
29.17	±	0.56	2135	fatty acid ester		5.19				+						
29.42	±	0.03	2149	fatty acid ester		6.54							++	∼		
31.20	±	0.07	2351	fatty acid ester	5.29								+		+	
31.34	±	0.03	2360	aldehyde		6.55	+	∼					+	+++		
31.53	±	0.03	2371	fatty acid ester	4.26		+						++			
31.55	±	0.12	2372	fatty acid ester	4.96	5.12									++	
31.84	±	0.05	2389	fatty acid ester	7.92		+						+		+	
31.89	±	0.07	2392	fatty acid ester	4.19				∼						+++	
32.19	±	0.46	2400	unknown	5.46								+		++	
33.02	±	0.04	2410	fatty acid ester		7.06			++	++	+++					
33.43	±	0.05	2486	hydrocarbon		6.00						∼		∼		++
34.04	±	0.04	2524	fatty acid ester	5.87	6.81					+++		+++	+	+	
34.56	±	0.06	2557	fatty acid ester	5.71		+++	+				+++	+		++	
35.04	±	0.04	2588	fatty acid ester		11.00	++	++					+			
35.19	±	0.07	2598	fatty acid ester	5.59	6.42							+		+++	
35.87	±	0.06	2542	unknown		5.90				+++	++				+	∼
37.70	±	0.01	2666	fatty acid ester		7.41	∼						++	++		
37.74	±	0.03	2669	unknown	4.11	6.44		++						+		∼
37.80	±	0.05	2673	unknown	6.05	8.77	++		+	+++	+++					
37.83	±	0.05	2675	unknown		7.54		+++				+	+			∼
37.91	±	0.03	2681	hydrocarbon	6.75	6.28	+		+		∼		++	++		
38.14	±	0.07	2697	fatty acid ester	5.57		+				+		+			
38.66	±	0.03	2734	unknown	4.05						∼				++	
38.69	±	0.04	2736	unknown		6.06							++			∼
38.95	±	0.09	2754	unknown	5.58		∼								+	
39.15	±	0.04	2768	unknown		5.88						+			+	+

#### (iii) Effect of life stage on chemical composition

As species-specific differences pointed towards a role during reproduction (e.g. pre-mating barrier), we screened chemical profiles of sexually mature and immature *O. ziama* frogs (36 collected from Vérè River, dry season). Among them, 10 were adults (female: *n* = 5, male: *n* = 5), 19 subadults (female: *n* = 10, male: *n* = 9) and 7 metamorphs (table 1). Subadults (and *vice versa* adults) lacked external sexual characteristics. Males usually develop vocal sacs and femoral glands at about SVL ≥ 38 mm, hence females were identified by their absence in individuals exceeding SVL ≥ 40 mm. Sex of smaller individuals was determined during dissection. In metamorphs (SVL ≤ 25 mm), sex could not be identified. Ordination separated individuals by life stage (adults, subadults and metamorphs) and sex (female, male and juvenile = metamorphs; figure 3*b*). However, group homogeneity was not given for sex (d.f. = 2, *F* = 11.01, *p* < 0.01) and life stages (d.f. = 2, *F* = 7.63, *p* < 0.01). Therefore, we reran the analysis, separating sexes according to life stage (male and female of adults and subadults individually), regrouping life stages into non-reproductive (subadults and metamorphs; grey dot and polygon in figure 3*b*) and reproductive stages (adults). Significant differences were found depending on sex (d.f. = 4, *F* = 2.29, *p* < 0.01) and reproductive status (d.f. = 1, *F* = 2.67, *p* < 0.01), confirmed by homogeneous group variances (reproductive stage: d.f. = 1, *F* = 0.90, *p* = 0.35; sex: d.f. = 4, *F* = 1.45, *p* = 0.24).

#### (iv) Effect of season and developmental status of strand

To screen for seasonal variations in the chemical composition (e.g. breeding season), we sampled additional strand excerpts from *O. arndti* in the wet season to compare them with the dry season samples. Furthermore, we categorized mandibular strands as ‘well-developed’ and ‘underdeveloped’. Moderately developed strands were excluded, resulting in 16 wet season samples (female *n* = 4, male *n* = 12) and 10 dry season samples (female *n* = 5, male *n* = 5). Coincidentally, all wet season females had well-developed strands, while in dry season females four out of five had underdeveloped strands (Barnard's test; *SS* = −2.40, *p* = 0.01). This bias was not evident in males (wet season: 7 out of 12 strands well-developed, dry season: three out of five; Barnard's test; *SS* = −0.06, *p* = 0.53). Variance homogeneity for both seasons was given for females (d.f. = 1, *F* = 1.01, *p* = 0.35) and males (d.f. = 1, *F* = 3.48, *p* = 0.08). Seasonal differences of the chemical composition were significant for females (d.f. = 1, *F* = 9.24, *p* = 0.01) but not for males (d.f. = 1, *F* = 0.77, *p* = 0.62). However, chemical composition of well- and underdeveloped strands differed significantly in males (d.f. = 1, *F* = 0.19, *p* < 0.01), and females (d.f. = 1, *F* = 3.96, *p* = 0.04; figure 3*c*). Variance homogeneity was given for well- and underdeveloped strands in females (d.f. = 1, *F* = 0.04, *p* = 0.86) and males (d.f. = 1, *F* = 0.86, *p* = 0.37).

#### (v) Effect of geographical distance

To test if the observed species-specific differences of chemical composition are consistent within a species or vary between populations, we compared frogs from different localities/populations. To exclude the effect of the strand's developmental status, we restricted this analysis to animals with well-developed stands. We analysed eight *O. smithi* female samples and nine samples from *O. arndti* males, each from three rivers across their respective distribution area (electronic supplementary material, figure S1). *Odontobatrachus smithi* samples originated from the rivers Naremba (*n* = 4), Ideta (*n* = 2) and Sigita (*n* = 2), collected in the dry season. The maximal longitudinal distance between the rivers was about 136 km. *Odontobatrachus arndti* samples were from the rivers Wolanda (*n* = 2), Mandey (*n* = 2) and Zougué (*n* = 5). Here the maximal longitudinal distance was about 6 km. However, these rivers were located on opposite slopes of the Nimba Mountains, and the rivers therefore are separated by a steep crest covered with high-altitude savannah, a habitat type that is not suitable for *Odontobatrachus*. Hence, the horizontal distance between the rivers (without leaving the forest) exceeded 20 km. For both *O. arndti* males (d.f. = 2, *F* = 1.86, *p* = 0.09) and *O. smithi* females (d.f. = 2, *F* = 1.51, *p* = 0.27), the PEMANOVA indicated no significant differences in group means from different localities. However, variance homogeneity was only given for *O. arndti* males (d.f. = 2, *F* = 1.17, *p* = 0.37), but not for the *O. smithi* females (d.f. = 2, *F* = 80.39, *p* < 0.01). We therefore performed Mantel tests on the dissimilarity matrices of the chemical fingerprint and the corresponding distance matrices for the two species. Neither *O. arndti* males (*r*^2^ *<* 0.01, *p* = 0.45)*,* nor *O. ziama* females (*r*^2^ *=* 0.04, *p* = 0.33)*,* showed a significant spatial influence on the chemical composition, thus confirming species-specific chemical composition.

## 4. Discussion

All five species and all sexes of the Odontobatrachidae family possess paired glandular strands hidden under the skin in their lower jaw. The strands produce sex- and species-specific chemical profiles that are composed of several different organic compound classes, mainly fatty acid esters with undetermined structure. These chemical profiles are absent from other tissues and vary between sexually mature and immature animals, coinciding with the developmental state of the strands. While these (chemical) properties clearly speak in favour of a signalling role for the strands, their anatomy does not support an exocrine function. Although there are anatomical peculiarities that may, theoretically allow an excretion—comparatively thin skin—the identity of the mandibular strand as a gland, as well as its function, remains to be clarified.

### (a) Fat tissue as signalling organ

The glandular strands comprised macrocytic white fat tissue, which in anurans is usually associated with intestinal (gonadal) fat bodies [38,39]. Additionally, cutaneous and subcutaneous fat tissue may appear evenly distributed along the body in various anurans, but concerted deposits have not been described [22,40]. Generally, white fat tissue is associated with energy storage, and it is plausible that the original function of the mandibular strands was, or partly still is, that of an energy storage. However, the fat tissue of the mandibular strands showed anatomical differences that may indicate a different function. First, and in contrast to subdermal fat tissue, the strand is associated with muscle tissue rather than skin. Second, the fat cells in the strand appeared larger than those in subdermal fat tissue (figure 2*f* and electronic supplementary material, figure S2A). Most importantly, even in the least developed strands, the fibroblasts and extracellular matrix of the strand and its characteristic chemical composition were still detectable (figures 2*e*,*f*, 3*f*, electronic supplementary material, figures S2 & S7). Subdermal fat tissue is usually fully resorbed when the energy storages are depleted (compare skin in figure 2*e*,*b*; [22,38]). Besides, energy storage and signalling function are not mutually exclusive, and fat tissue is lately acknowledged for its fundamental role in (endocrine) signalling in vertebrates [41]. Macrocytic fat tissue mainly contains triglycerides and it is conceivable that these may serve as a template for the fatty acid esters identified in the strands. At least in insects, these storage fats serve as precursor molecules for pheromone production and are associated with signalling tissues [42–44], thus a similar function may be attributed within our study species.

### (b) Fatty acid esters as potential signalling molecules

Mass spectra pointed toward fatty acid derivatives, mainly esters, as main components in the mandibular strands. Classification analysis (random forests) indicated the relevance of these compounds for the species and sex-specific fingerprint. Nonetheless, we have no proof that these fingerprints comprise an actual signal. However, the fatty acid esters were specific to the strand tissue, hence we can rule out that they represent unspecific cellular components (e.g. parts of the cell membrane; electronic supplementary material, figures S4, S5 & S6). Additionally, fatty acid esters are not primary metabolites required in homeostasis, thus our best explanation is that they are mediating, secondary metabolites [45,46]. In respect to the species-specificity, the most parsimonious explanation is that the strand-specific compounds either comprise a signal themselves or are at least directly connected to it, be it as educts or precursor molecules [47]. In fact, fatty acid esters have already been identified in the femoral glands of mantellid frogs and the gular patches of several hyperoliids, two macro-glands known to produce volatile species-specific cues. However, fatty acid esters themselves have not yet been associated with a signalling function in these cases (particularly because of their lower volatility in comparison to other components from these breeding glands; [5,16]). Fatty acid esters however, are common pheromones in insects and hence are suitable signalling molecules [48,49].

### (c) A breeding—related function

Both our anatomic findings and the chemical results suggest that the markedness of the mandibular strand changes throughout the reproductive cycle of both sexes. The macroscopic and microscopic differences between well and underdeveloped strands (and all intermediate stages) can be explained by storing lipids in the vesicle of the macro-vesicular fat cells [39,50]. This coincided with a detectable change in the chemical fingerprint, likely an effect of lowered concentrations of the same compounds (figure 3*b*; electronic supplementary material, figure S9). These aspects support a breeding-related function of the mandibular strands, which, together with the chemical properties of its product (volatile, sex- and species-specificity) tempts us to conclude that the mandibular strand comprises a novel type of breeding gland. In contrast, the strand's anatomy differs vastly from that of any other sexually dimorphic breeding gland [51,52]. First, it exists in both sexes and is not a skin-derived, superficial structure. Second, the release mechanism is not exocrine as the tissue lacks (among other criteria) interlacing musculature and an intercalary duct.

Nevertheless, we believe it is most likely that the strand-specific components are emitted via, or at the level of, the external vocal sac in males and the post-mandibular patch in females. This supposition is mainly based on the observation that these two skin sections are directly connected to the strand and were the only external tissues that contained traces of the strand-specific compounds. In addition, in both sexes these skin sections showed a thinned-out epidermis and a lack of epidermal skin glands, features that have been associated with increased secretions in the sternal skin region of male *Breviceps gibbosus* (figure 2*e*; electronic supplementary material, figure S2B; [53]). The bulging of the external vocal sacs in males and the widening of the skin tubercles in the females' post-mandibular patch during the reproductive phase may additionally increase the permeability, particularly for lipophilic substances (figure 1*b*; [54,55]). Strand contents may therefore be secreted into the strands’ extracellular matrix, and from there can probably be pushed forwards when neighbouring muscles contract. Exploitation of mechanical work by neighbouring structures is a common physiological principle, including transportation of glandular secretions [56–58]. A mechanical dependency could also explain the identified anatomical differences between male and female frogs (e.g. episternal strand connected to interhyoid muscle only in females). While in males, the delivery of the strand's secretion could be linked to movements of the inner vocal sac (e.g. inflating), females would need a more direct connection between muscles and strand to deliver any secretions (e.g. when intermandibular and interhyoid muscles contract). In *Odontobatrachus* both sexes call (Schäfer *et al.* 2018, unpubl. data), and we thus believe that the secretion of the strand product is associated with active movement of the buccal pump [37]. However, at this stage this mechanism is hypothetical and requires further investigation, particularly ultrastructural analysis of the strand-skin interface. Nevertheless, chemical communication is by no means restricted to particular structures or sex, as any cue may evolve into a signal as long as it conveys meaningful information and this is especially true for chemical ones [59,60].

### (d) Chemical communication in the Odontobatrachidae?

Our analysis focused on volatile substances only. Also, we have not isolated individual substances and did not test their respective effects of on the behaviour of frogs. The chemical properties and the comportment of the strand, however, point towards signalling molecules and a breeding-related role in the five cryptic species. Unfortunately, the breeding behaviour of the Odontobatrachidae is not yet well understood. Some observations indicate why additional (chemical) signals could be of importance in saber-toothed frogs. All species are torrent specialists, and first data of their breeding ecology showed that eggs are laid in narrow crevices that are not accessible for an amplectant pair [61]. Consequently, tactile signals (or the exchange of signalling molecules via the skin) are not available to coordinate oviposition and fertilization, requiring another mode of communication. Additionally, there is evidence for violent fighting with potentially fatal outcome, mainly among males, but suspectedly also among females [61]. The chemical signal may therefore serve as an honest signal to assess fighting ability, where calling may convey deceptive information [59,62,63]. Alternatively, the signal does not have its own specialized function, but simply increases the perceptibility of the individuals, particularly as we hypothesize that the emitting of the signal is closely related to calling. Multimodality is recently acknowledged to be widely used among amphibians [64,65]. It is plausible that an additional signal might help the genus to communicate through their noisy habitat along waterfalls and cascades. Several other phylogenetically unrelated torrent frogs living in similarly noisy conditions to *Odontobatrachus* face the same constraints of vocal communication and thus accumulate an outstanding variety of adaptations, such as ultrasonic communication [66–68], or food flagging [69].

## 5. Conclusion

Our results suggest that the mandibular strand in the Odontobatrachidae comprises a thus far overlooked, breeding-related structure. Though the signalling molecules can not yet be exactly identified, and the suggested emitting mechanism is based on histology and anatomy only, the chemical signature of the strand supports its role in the breeding behaviour of the five *Odontobatrachus* species. We hypothesize that the release of the signal is associated with calling behaviour, due to the volatility of the chemical profiles, as well as the placement and sex-specific differences in the strand's anatomy. While we do not yet know the exact function of the signal, we assume that an olfactory signal, in addition to calls, may facilitate communication through the noisy torrents that the Odontobatrachidae inhabit. Our results thus highlight that chemical signalling in anurans may not be restricted to sexually dimorphic, apparent skin glands.

## Data Availability

Original data and fully reproducible code are available under: https://doi.org/10.5061/dryad.t1g1jwt8f [33]. Supplementary material is available online [70].
